# Bio-Fabricated Aluminum Oxide Nanoparticles Derived from Waste Pharmaceutical Packages: Insight into Characterization and Applications

**DOI:** 10.3390/biom15070984

**Published:** 2025-07-10

**Authors:** Jamilah M. Al-Ahmari, Reem M. Alghanmi, Ragaa A. Hamouda

**Affiliations:** 1 Department of Chemistry, College of Science, University of Jeddah, P.O. Box 80327, Jeddah 21589, Saudi Arabia; rmalghanmi@uj.edu.sa; 2 Department of Applied Radiologic Technology, College of Applied Medical Sciences, University of Jeddah, Jeddah 23218, Saudi Arabia; 3 Microbial Biotechnology Department, Genetic Engineering and Biotechnology Research Institute, University of Sadat City, Sadat City 32897, Egypt

**Keywords:** aluminum oxide, pharmaceutical packages, marine algae, adsorption, Congo red dye

## Abstract

This study examines the environmental challenges posed by azo-dye pollutants and aluminum industrial waste. Aluminum oxide nanoparticles (P/Al_2_O_3_-NPs) were produced using a green method that utilized pharmaceutical packaging waste as an aluminum source and marine algae extract (*Padina pavonica*) as reducing and stabilizing agents and that was characterized by XRD, EDX, SEM, TEM, and zeta potential. Batch biosorption studies were performed to assess the effectiveness of P/Al_2_O_3_-NPs in removing CR dye from aqueous solutions. The results demonstrate that the particle sizes range from 58.63 to 86.70 nm and morphologies vary from spherical to elliptical. FTIR analysis revealed Al–O lattice vibrations at 988 and 570 cm^−1^. The nanoparticles displayed a negative surface charge (−13 mV) and a pH_zpc_ of 4.8. Adsorption experiments optimized parameters for CR dye removal, achieving 97.81% efficiency under native pH (6.95), with a dye concentration of 30 mg/L, an adsorbent dosage of 0.1 g/L, and a contact time of 30 min. Thermodynamic studies confirmed that the process is exothermic and spontaneous. Kinetic data fit well with the pseudo-second-order model, while equilibrium data aligned with the Langmuir isotherm. The adsorption mechanism involved van der Waals forces, hydrogen bonding, and π–π interactions, as supported by the influence of pH, isotherm data, and FTIR spectra. Overall, the study demonstrates the potential of eco-friendly P/Al_2_O_3_-NPs to efficiently remove CR dye from aqueous solutions.

## 1. Introduction

As a result of increasing urbanization and population worldwide, the industrial revolution emerged to meet the needs of human societies for goods and basic life requirements, as the world was constantly seeking to increase productivity. At the same time, it was not fully prepared to deal with the enormous amount of waste generated, which plays a major prominent role in the global pollution problem. Among the pollutants of public health and environmental concern due to their toxicity are nuclear waste, heavy metals, azo dyes, hydrocarbons, pesticides, and greenhouse gases [1]. In the pharmaceutical sector, blister packs are now the most popular packaging for tablets and capsules [2]. A significant portion of municipal solid waste consists of packaging waste, which has led to growing environmental concerns [3]. The amount of waste generated by pharmaceutical packaging has steadily increased over time due to the growing number of uses in daily life [4].

Aluminum is one of the most vital metals in the world, second only to steel, with 68.41 million tons of primary production in 2022. It is also among the most recycled metals because the total energy used in the aluminum recovery procedure is 95% less than that required for primary aluminum manufacture [5]. As a method of environmental remediation, the discarding of waste products is frequently accomplished through either landfilling or incineration [6]. Aluminum-containing waste can release gases, create leachates, and generate adverse thermal effects when disposed of in landfills. These processes account for approximately 3% of global greenhouse gas emissions [7]. Therefore, developing a novel, sustainable approach for efficiently producing pure alumina nanoparticles from solid waste is essential. Nanoparticles have gained significant attention worldwide due to their unique characteristics and diverse applications. In particular, metal and metal oxide nanoparticles present a promising opportunity in the water treatment industry for effectively removing various contaminants. Biological synthesis provides an eco-friendly, cost-effective, and sustainable substitute to conventional synthetic methods, addressing environmental concerns and simplifying production [8]. Many biological resources have been used to produce metal and metal oxide nanoparticles, including bacteria, fungi, algae, and plants [9]. *Padina pavonica*, a brown marine alga rich in biologically active compounds such as sulfated polysaccharides and polyphenols, has emerged as an eco-friendly platform for the green synthesis of metal and metal oxide nanoparticles. Its aqueous extract acts as both a reducing and stabilizing agent, removing the need for hazardous chemicals typically used in traditional nanoparticle production. Recent studies have demonstrated the successful synthesis of zinc oxide (ZnO) [10], iron oxide (Fe_3_O_4_) [11], and silver (Ag) nanoparticles [12] using *Padina pavonica* extract, highlighting its potential for environmental applications. The metal and metal oxide nanoparticles produced by *Padina pavonica* extract have demonstrated promise in pollutant removal, dye degradation, and antimicrobial treatment [10,11,12]. This green approach offers a cost-effective and sustainable method for developing nanomaterials for water purification and pollution control. However, few studies have been available on recycling solid waste to produce metal oxide nanoparticles using algae as a biological reducing and stabilizing agent. Further research is needed to investigate the production of aluminum oxide nanoparticles by marine algae and their potential applications in water treatment.

**Scheme 1:**
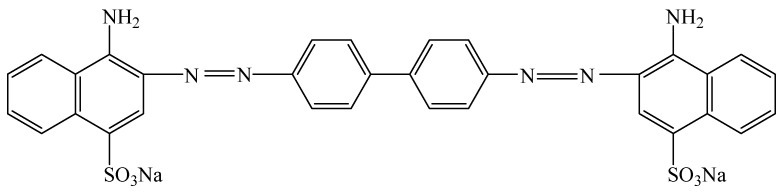
Chemical structure of CR dye.

Many methods have addressed recycled aluminum from waste, such as melting, sorting, and thermal treatments [7]. Aluminum foil can be used as a precursor for manufacturing eco-friendly products [13]. Numerous studies report the use of aluminum foil as a precursor for synthesizing green aluminum oxide nanoparticles utilizing various plants as reducing and stabilizing agents, such as the leaf extract of *Muntingia calabura* [14], grapefruit extract [15], and the leaf extract of Rosa [16]. Marine algae are rich sources of bioactive compounds, including phenolic compounds, steroids, saponins, tannins, reducing sugars, and carbohydrates [17]. *Sargassum ilicifolium*, a marine alga, was used to produce alpha aluminum oxide nanoparticles [18]. Alumina nanoparticles exhibit high efficiency in removing cationic dyes [19]. Additionally, alumina nanoparticles offer new potential for reliable and economically sustainable water treatment to eliminate anionic dyes from aqueous solutions [20]. Congo red (CR) is a synthetic, benzidine-based anionic azo dye commonly used in the textile, paper, printing, and plastic industries due to its bright colors and resistance to fading [21]. Structurally, it features aromatic rings connected by azo (–N=N–) groups and sulfonic acid substituents that make it water-soluble (Scheme 1). While industrially useful, CR raises significant environmental and public health risks. It is highly stable in water, resistant to biodegradation, and toxic to aquatic life [22]. More critically, CR can undergo reductive cleavage in anaerobic conditions to produce benzidine, a recognized human carcinogen and mutagen. Long-term exposure to CR-contaminated water has been associated with cytotoxic, genotoxic, and teratogenic effects [23,24]. Considering these physicochemical properties and associated health hazards, it is crucial to explore effective methods for removing CR from aquatic environments. Tackling its persistence and toxicity through adsorption or degradation is vital for protecting both environmental and public health.

Given the environmental burden posed by pharmaceutical packaging waste and the increasing demand for sustainable water treatment solutions, this study aims to develop an eco-friendly and efficient method for fabricating aluminum oxide nanoparticles (Al_2_O_3_-NPs) by recycling pharmaceutical packaging waste using marine algae (*Padina pavonica*) as a biological reducing and stabilizing agent. This biogenic approach offers a green fabrication pathway that minimizes chemical use and promotes resource recovery from solid waste. The resulting biogenic P/Al_2_O_3_-NPs were thoroughly characterized to assess their physicochemical properties and surface functionalities using pH zero point charge (pHzpc), FTIR, EDX-SEM, XRD, TEM, and zeta potential analysis. Subsequently, their effectiveness in removing anionic dyes, specifically Congo red (CR), from aqueous solutions was evaluated under varying conditions. Thermodynamic, kinetic, and isothermal adsorption models were applied to understand the adsorption mechanism and governing interactions. The study provides evidence for the promising application of biogenic alumina nanoparticles synthesized from pharmaceutical waste as an effective and sustainable material for water remediation. By integrating waste valorization with green nanotechnology, this work contributes to circular economy practices and sustainable environmental engineering.

## 2. Results and Discussion

### 2.1. Characterization of Biogenic P/Al_2_O_3_-NPs

#### 2.1.1. pH Zero Point Charge (pH_zpc_)

**Figure 1:**
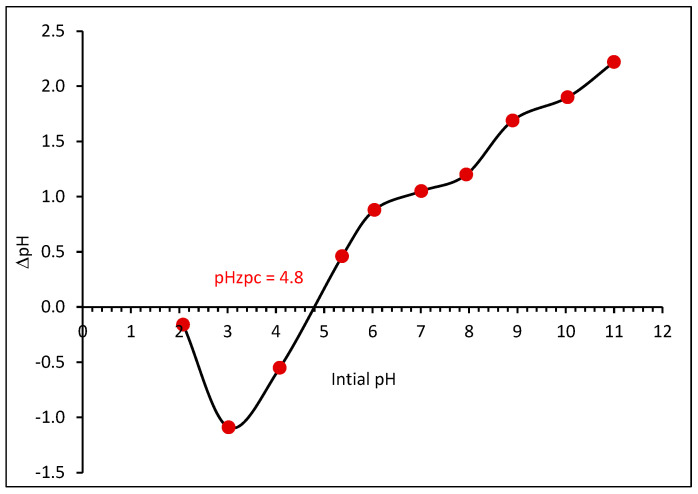
pH_zpc_ plot for biogenic P/Al_2_O_3_-NPs derived from *Padina pavonica* using waste pharmaceutical packages as precursors.

To characterize the surface charge of the biogenic P/Al_2_O_3_-NPs derived from *P. pavonica* using waste pharmaceutical packages as precursors, the pH at the point of zero charge was discovered using the solid addition method [25]. The pH_zpc_ of an adsorbent is an important characteristic for predicting how the adsorbent surface behaves and for establishing the pH value at which the surface becomes electrically neutral [26]. Figure 1 shows the plot of ΔpH versus the initial pH for the P/Al_2_O_3_-NPs suspension, where the pH_zpc_ is indicated by the point where ΔpH = 0, and it was recorded as 4.8. Below the pH_zpc_ value, the P/Al_2_O_3_-NPs surface is net positively charged, while above that value, the net surface is negatively charged [26]. The measured pH_zpc_ for the biogenic P/Al_2_O_3_-NPs is substantially lower than the values of ~7.0–8.0 typically reported for Al_2_O_3_ synthesized by conventional methods [20,26]. The lower pH_zpc_ in this work likely reflects the influence of the biogenic process. Algal extracts contain abundant hydroxyl, carbonyl, and other acidic functional groups (e.g., polysaccharides and phenolics) that can bind to the alumina surface during reduction [10,27]. Koopi and Buazar (2018) noted that hydroxyl and carbonyl groups from brown algae extract interact with Al^3+^ to form Al_2_O_3_ NPs [18]; such functionalization tends to lower the net surface pK_a_.

#### 2.1.2. FTIR Spectra

**Figure 2:**
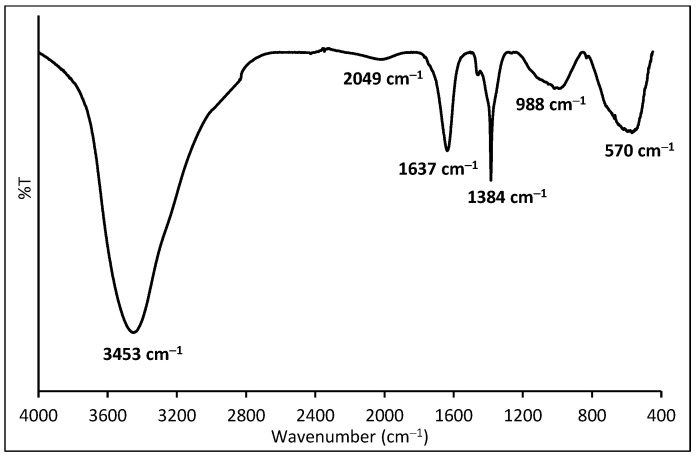
FTIR analysis of biogenic P/Al_2_O_3_-NPs derived from *Padina pavonica* using waste pharmaceutical packages as precursors.

The FTIR spectrum of the biogenic P/Al_2_O_3_-NPs derived from *P. pavonica* using waste pharmaceutical packages as precursors is shown in Figure 2. The spectrum indicates the presence of several characteristic absorption peaks at 3420, 2049, 1637, 1459, 1384, 988, and 540 cm^−1^. The broad peak at 3420 cm^−1^ is assigned to the O–H stretching, and the peak at 1637 cm^−1^ is assigned to the bending of H–O–H of the surface hydroxyl group and adsorbed water [20,28]. Recent investigations on γ-Al_2_O_3_ have confirmed the presence of surface –OH groups, which provide hydrophilicity and reactive sites on the nanoparticles, as these peaks are characteristics of alumina nanoparticles [29,30]. The peaks observed at 2049, 1459, and 1384 cm^−1^ are attributed to C≡C stretching, aromatic C=C stretching, and C–H bending vibrations, respectively. These peaks are likely associated with the organic constituents present in the *Padina pavonica* extract [10]. Two noticeable peaks at 988 cm^−1^ and 570 cm^−1^, just below 1200 cm^−1^, are attributed to vibrations in the Al–O lattice (Figure 2). A strong absorption at 570 cm^−1^ arises from the stretching movements of octahedral AlO_6_, while the band at 988 cm^−1^ relates to faster Al–O (or Al–O–H) vibrations [31]. These results align with the γ-Al_2_O_3_ signals that have been published at ~840 cm^−1^ (tetrahedral) and ~560 cm^−1^ (octahedral) [32]. This suggests that P/Al_2_O_3_-NPs derived from *P. pavonica* using waste pharmaceutical packages assume a transitional alumina structure with established oxide networks.

#### 2.1.3. Energy-Dispersive X-Ray Spectroscopy

**Figure 3:**
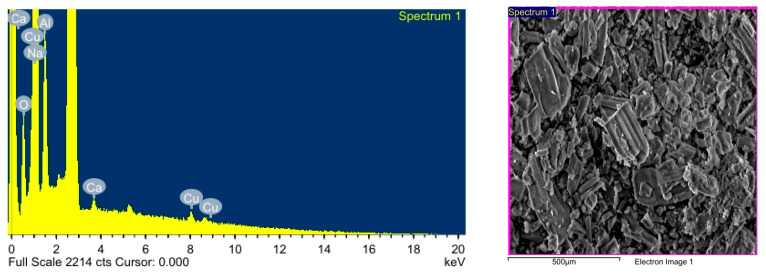
EDX analysis and SEM image of biogenic P/Al_2_O_3_-NPs derived from *P. pavonica* using waste pharmaceutical packages as precursors.

Figure 3 and Table 1 display the energy-dispersive X-ray spectrophotometry (EDX) analysis and scanning electron microscope (SEM) images of P/Al_2_O_3_-NPs utilizing waste pharmaceutical packages as precursors. The findings reveal five metals, O, Na, Al, Ca, and Cu, with weight percentages of 13.38, 72.69, 11.00, 0.68, and 2.25, respectively. These results indicate that the nanoparticles are Al_2_O_3_ nanoparticles due to the presence of oxygen and aluminum elements. The EDX measurements of Al_2_O_3_ confirm the presence of Al and O peaks [33].

#### 2.1.4. Zeta Potential Analysis

**Figure 4:**
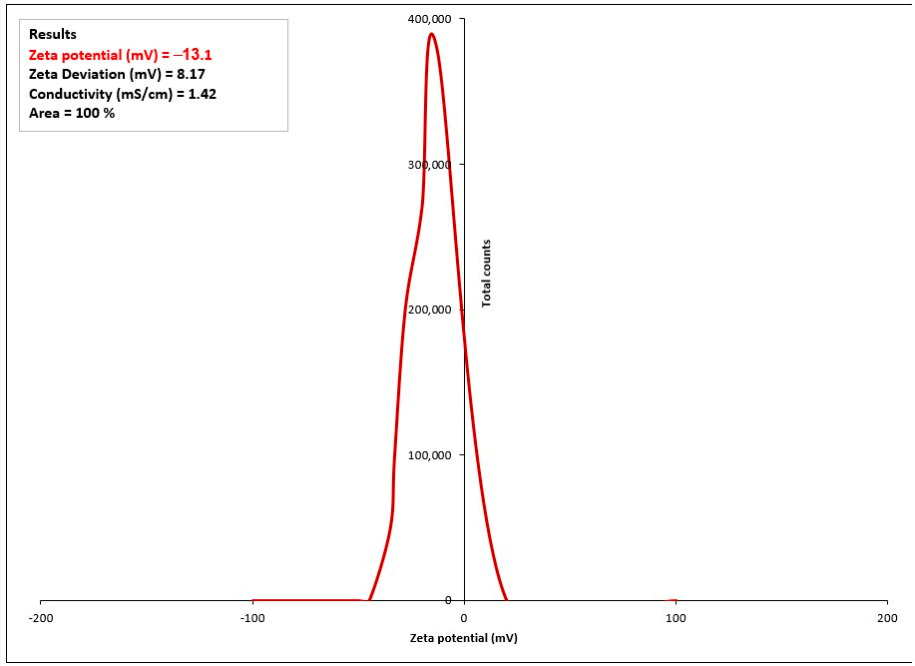
Zeta potential analysis of biogenic P/Al_2_O_3_-NPs derived from *P. pavonica* using waste pharmaceutical packages as precursors.

The zeta potential is an analysis method that determines the number of electric charges on the surfaces of nanoparticles [34]. The results in Figure 4 demonstrate the zeta potential value of biogenic P/Al_2_O_3_-NPs in negative charges (−13 mV), which indicates that the surface has a negative charge and moderate electrostatic stability. Al_2_O_3_ nanoparticles kept their negative surface charge over time [35]. Similar green-fabricated aluminum oxide nanoparticles, such as those fabricated with *Sargassum* sp. [18] and Lyngbya majuscula [36], show zeta potential values between −10 and −15 mV, indicating that algal polysaccharides and phenolic compounds are coating the oxide surface [18,36]. The presence of high amounts of Na may be due to an increase in pH values. When the pH value is 9 (electrolyte pH value > IEP), the zeta potential of α-Al_2_O_3_ is −16.69 mV [37].

#### 2.1.5. X-Ray Diffraction Analysis

**Figure 5:**
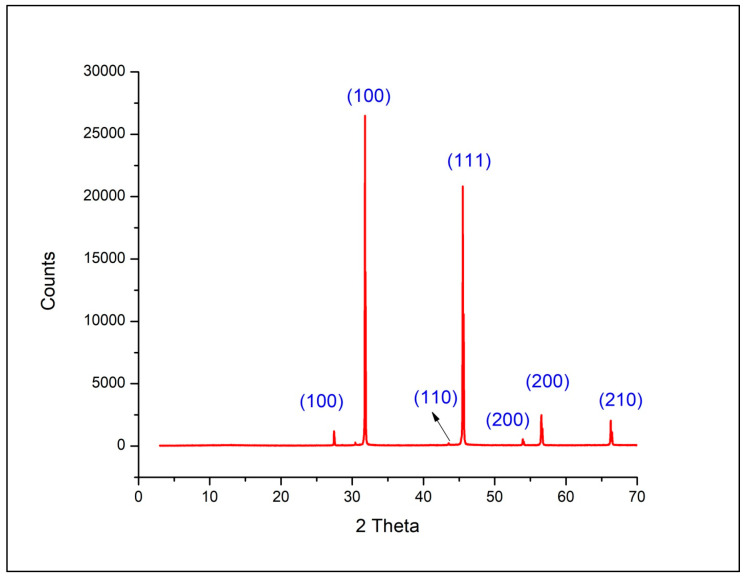
X-ray diffraction patterns of biogenic P/Al_2_O_3_-NPs derived from *P. pavonica* using waste pharmaceutical packages as precursors.

The results in Figure 5 and Table 2 demonstrate the X-ray diffraction patterns of biogenic P/Al_2_O_3_-NPs derived from *P. pavonica* using waste pharmaceutical packages as precursors. The results display sharp diffraction Bragg peaks located at 2Theta 27.453°, 30.433°, 31.787°, 43.527°, 45.519°, 53.932°, 56.537°, and 66.28°, which indexed to (100), (100), (100), (110), (111), (200), (200), and (210) plane, respectively. The sharpness of these reflections confirms the high crystallinity of the P/Al_2_O_3_-NPs, as shown in Figure 4. The most intense peaks at 2Theta are equal to 31.787° and 45.519°, reach relative intensities of 100% and 87.4%, respectively, and correspond to average nanoparticle sizes of 82.89 and 86.45 nm.

#### 2.1.6. TEM Analysis

**Figure 6:**
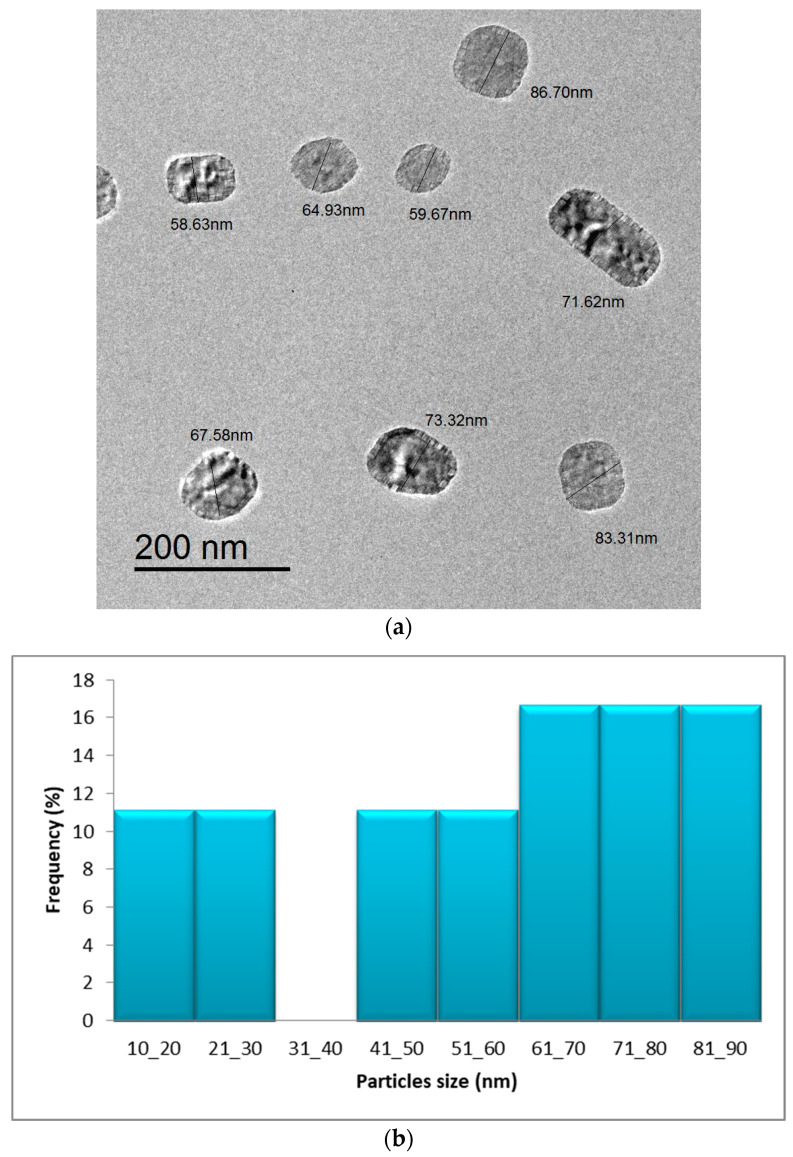
(**a**) TEM image of biogenic P/Al_2_O_3_-NPs derived from *P. pavonica* using waste pharmaceutical packages as precursors. (**b**) Particle size distribution of biogenic P/Al_2_O_3_-NPs derived from *P. pavonica* using waste pharmaceutical packages as precursors.

The results in Figure 6 demonstrate the size and shape of P/Al_2_O_3_-NPs derived from *P. pavonica* using waste pharmaceutical packages as precursors. The P/AL_2_O_3_-NPs diameter ranged from 58.63 to 86.70 nm, with a spherical to ellipsoidal shape and high crystallinity. The size distribution suggests moderate polydispersity.

The results in Figure 6b demonstrate that the particle sizes ranged from 10 to 90 nm. The frequency of particle sizes of P/Al_2_O_3_-NPs has two distinct groups: one in the smaller size ranges (10–30 nm and 41–60 nm), with a range frequency of 11%, and a larger concentration in the higher size ranges (61–90 nm), with a range frequency of 17%. The majority of particles are found in the larger size ranges (61–90 nm). This suggests a uniform distribution of particles across two size ranges, implying there is no dominant particle size within this interval. The particles are evenly distributed across the specified size ranges.

### 2.2. Adsorption Study

#### 2.2.1. Effect of pH

**Figure 7:**
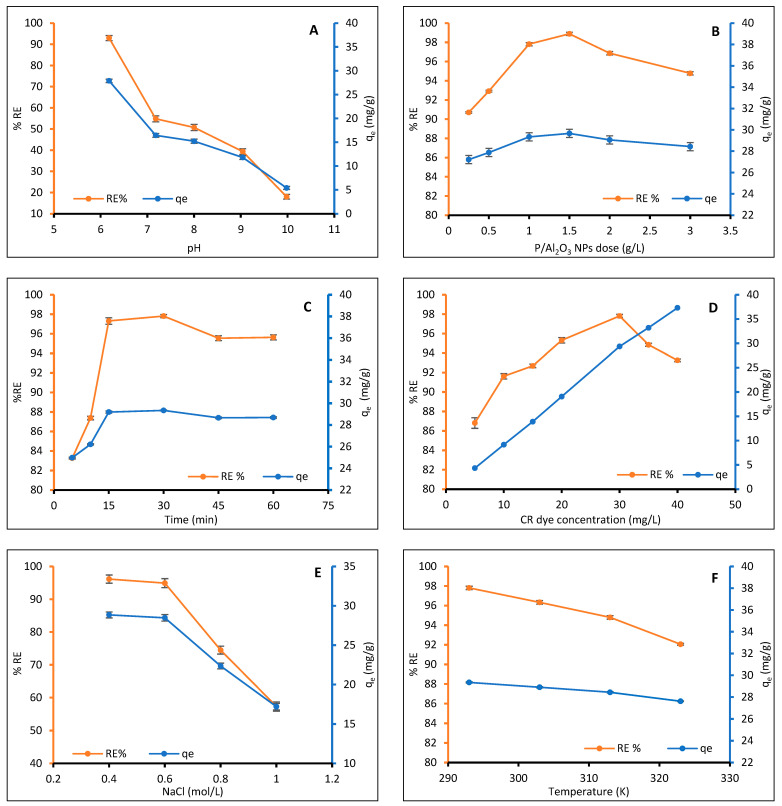
Effect of different factors on the percentage removal of CR dye and the adsorption capacity of biogenic P/Al_2_O_3_-NPs derived from *P. pavonica* using waste pharmaceutical packages as precursors: (**A**) pH, (**B**) adsorbent dose, (**C**) contact time, (**D**) initial concentrations of CR dye, (**E**) ionic strength, (**F**) temperature.

The pH of the solution is a critical factor in the dye adsorption process. It significantly affects the electrical charge of the adsorbent surface and the degree of ionization of the dye molecules that function as adsorbate material. This factor influences the dissociation process of various functional groups on the active sites of the surface of the adsorbent [27]. Figure 7A illustrates the effect of pH on the removal percentage (RE%) and adsorption capacity (q_e_) of CR dye using P/Al_2_O_3_-NPs as an adsorbent. The adsorption efficiency recorded exceptionally high values of RE% and q_e_ at pH 6.0 (93.0% and 27.9 mg/g, respectively). It declined across the investigated range, reaching very low values (18.0% and 5.41 mg/g) as pH increased, which corresponds to rising solution alkalinity (pH = 9.98). The effect of pH variations can be explained by considering the pH_zpc_ value of 4.8 (Figure 7A), which provides a clearer understanding of the electrostatic interactions at the adsorbent–adsorbate interface [18]. The surface of the nanoparticles carries a negative charge above the pH_zpc_ value and a positive charge below it. Since CR dye is an azo anionic dye with a pK_a_ of 4.1, its removal from solutions is favored at an acidic pH value, as this enhances the likelihood of significant electrostatic attraction between the negatively charged dye molecules and the positively charged surface of the nanoparticles [38].

The low adsorption efficiency of P/Al_2_O_3_-NPs in high-pH media can be attributed to two factors. First, the surface of the NPs is primarily negatively charged, which hinders the electrostatic attraction between them and the negatively charged dye molecules. In other words, the significant reduction in the dye removal may be due to the presence of repulsive forces between the surface of the nanoscale and the deprotonated dye molecules [39]. Another reason that cannot be overlooked is that at a high pH value, the positively charged interface solution decreases. The concentration of OH^−^ ions will gradually increase, competing with anionic dye molecules to access and occupy the remaining positively active sites on the surface of the nanocomposite [39,40]. On the other hand, the above effects may cooperate in more alkaline solutions, with a pH range of 8 to 9.98, where the adsorption capacity and the dye removal percentage are recorded to decrease sharply. The same behavior was reported for the adsorption of CR dye onto different nanoparticle adsorbents [41]. Also, Patel et al. recorded low removal of CR dye at pH = 10 when using iron oxide NPs fabricated by *Acacia jacquemontii* [42]. Similarly, Al-Ghamdi et al. indicated that the adsorption capacity of CR on MoS_2_ nanoparticles decreased with increasing pH due to repulsive electrostatic interactions at high pH levels [40]. Finally, this study highlights the pivotal role of pH in regulating the surface charge of both the adsorbent and the dye and, thus, the overall adsorption performance. Since the maximum removal was achieved at the native pH of the dye (6.95), all subsequent experiments were carried out at this pH.

#### 2.2.2. Effect of P/Al_2_O_3_-NPs Dosage

The results of studying the effect of P/Al_2_O_3_-NPs dosage are depicted in Figure 7B. The results indicate that the removal efficiency of P/Al_2_O_3_-NPs fabricated using waste pharmaceutical packages as precursors continued to increase with the dose of the NPs until reaching 1.5 g/L, at which point the removal percentage and adsorption capacity were 98.89% and 29.67 mg/g, respectively. Subsequently, a decline in value was noted at higher dosages, although the removal rates remain satisfactory. It is possible to assert that the increase in adsorption that occurs in conjunction with the increase in the dose of adsorbent is because the number of active sites that are available increases, as well as the opportunities for dye molecules to adsorb in the presence of a constant concentration of dye solution. This means that the removal rate per unit mass is increased. However, as the quantity of adsorbent material increases until it reaches values of 2.0 and 3.0 g/L, there is a corresponding decrease in the value of removal and adsorption capacity. This suggests that there is a decrease in the rate of mutual interaction between the dye molecules and the active sites that are easily accessible. This behavior can be attributed to the fact that, at a constant volume and concentration of the dye solution, an increase in the adsorbent dose will result in more unsaturated adsorption active sites. Another reason is the accumulation or overlapping of nanoparticle material resulting from a high dosage of adsorbent. This accumulation diminishes the overall accessible surface area for adsorption processes, extending the diffusional pathway [43]. Consistent with this, a study published in 2024 reported that high adsorbent dosages caused particle agglomeration, resulting in a reduction of available surface area and an elongation of the pollutant’s diffusion path within the sorbent material [44]. Taking into account the adsorption capacity of P/Al_2_O_3_-NPs of 29.34 mg/g, a 1.0 g/L dosage was selected for further experiments.

#### 2.2.3. Effect of Contact Time

Among the most influential parameters in the adsorption process is the contact time between the adsorbent and the adsorbate solution, which is essential for achieving maximum removal efficiencies. The effect of contact time on the adsorption of CR dye onto biogenic P/Al_2_O_3_-NPs derived from P. *pavonica* using waste pharmaceutical packages as precursors is illustrated in Figure 7C. During the early stages of the adsorption process, most of the removal processes reached their peak, and as time progressed, the reaction rate slowed down (Figure 7C). The adsorption rate of CR dye onto P/Al_2_O_3_-NPs increased as the removal percentage rose from 92.35% to 97.81%, with an increase in contact time from 5.0 to 30 min. Additionally, the adsorption capacity increased from 25.0 mg/g to 29.3 mg/g as the contact time was extended from 5 min to 30 min. It is evident from Figure 7C that the rate of the adsorption process was initially rapid until it reached 30 min, after which it slowed down. This is attributed to the abundant availability of active sites on the surface of P/Al_2_O_3_-NPs, which are readily available for the adsorption process. All of these conditions enable the quick attachment of dye molecules. However, as time progresses and adsorption continues, more and more CR dye particles are adsorbed, leading to a decrease in unoccupied surface sites, which results in a slowdown of the dye removal rate [45]. The same behavior was observed for removing various dyes, including Safranin, methylene green, Congo red, and methyl orange, by Kaolinite Nanotubes (KNTs), which displayed segmental adsorption curves. The initial steep slopes indicated rapid dye uptake, followed by gradual increases that led to saturation, and after that period, the amount of removal decreased [46]. A similar observation has been reported by Banerjee et al. (2019) for the adsorption of the anionic dye orange G onto alumina NPs, which were synthesized using the sol–gel precipitation method [20].

To be more specific, the adsorbent is responsible for removing CR molecules through a three-step process: (1) the movement of the CR molecules from the bulk of the solution to the surface of the biogenic P/Al_2_O_3_-NPs; (2) the diffusion of the CR molecules through the boundary layer to the adsorbent’s surface; and (3) the intraparticle diffusion of CR molecules within the interior pores of the adsorbent particle. Additionally, several factors can affect the resistivity of the boundary layer, including the rate of adsorption and the increase in contact time [47]. Therefore, it can be stated that the removal rate is primarily governed by the transport of dye molecules from the external active site to the internal active site of adsorbent particles [48]. According to a recent investigation, the optimal time for maximum dye removal was found to be half an hour, at which point equilibrium was completely achieved. Pal and his co-workers recorded results similar to the current study, where the nanocomposite demonstrated rapid and superior adsorption efficiency of RB dye within 40 min and CR dye within 30 min from the aqueous environment [49]. Likewise, Firmino and colleagues found that the NiFe_2_O_4_ fibers exhibited a high CR removal efficiency of ~97% after only 30 min [50]. In light of this, the contact time in the current study was determined to be 30 min.

#### 2.2.4. Effect of Initial Concentration of CR Dye

The effect of changing the initial concentration of the CR solution on the efficiency of the adsorption process was investigated. The results depicted in Figure 7D show an increase in the removal percentage and adsorption capacity, reaching their highest at 30 mg/L (97.81% and 29.34 mg/g, respectively). Beyond this point, a slight decrease was observed, with values remaining nearly constant as the concentration increased to 50 mg/L. The observed increase in uptake in dilute solutions can be attributed to the presence of unoccupied and unsaturated active sites on the surface of P/Al_2_O_3_-NPs relative to the total amount of dye molecules, which facilitates the adsorption process until it peaks at a concentration of 30 mg/L. Afterward, raising the dye concentration with a defined number of active sites of P/Al_2_O_3_-NPs will result in molecular competition and hinder accessibility. In contrast, most sites will become saturated simultaneously, which clarifies the slight stability observed at increased concentrations. To put it another way, a specific quantity of nanocomposite has the capability of adsorbing a specific quantity of dye molecules, which indicates that the percentage of CR removal is dependent on the initial concentration. Similarly, it was reported that the increase in the concentration of dye molecules exceeded the number of available sites on the surface of the nanomaterial NiFe_2_O_4_, leading to the saturation of these sites and stabilization of the adsorption capacity [50].

#### 2.2.5. Effect of Ionic Strength

Industrial wastewater is widely recognized to contain various additives, such as salts and surfactants, during dye-utilizing processes. These substances may hinder or accelerate the treatment process, depending on their impact on the interaction between the adsorbent and the dye molecules in the solution [51]. The concept of ionic strength refers to the concentration of ions in the solution, which actively influences the adsorption behavior of dye molecules. As CR dye is anionic, the adsorption efficiency is expected to be highly sensitive to the ionic composition of the surrounding medium. The results in Figure 7E demonstrate a pronounced and dramatic decline in dye adsorption as the concentration of NaCl solution increases. This can be ascribed to the influence of the negative chloride ion on the adsorption mechanism of anionic dye. At low chlorine anion concentrations, the adsorption of the dye remains dominant, even if it is lower than in the absence of NaCl solution, due to its abundance compared to the chloride concentration. On the other hand, while the concentration of NaCl in the solution increases, the competition between the two anions for access to available active surface sites also increases. Given the expectation of a preference from the P/Al_2_O_3_-NPs surface for the chloride ion, that ion takes over the surface. An additional factor that must be considered is the size of the ions. Relative to the dye ion, the chloride ion is diminutive and migrates more swiftly to bind with active sites. The performance of untreated nanodiamonds (UNDs) and thermally oxidized nanodiamonds (ONDs), used for the adsorption of methyl orange (MO) from aqueous media, was tested in the presence of NaCl and KCl salts, which showed similar decreases in the removal efficiency [52]. Furthermore, as the ionic strength of the solution increases, a reduction in the thickness of the double electrical layer surrounding the molecules will be expected, leading to a diminished electrostatic force between the adsorbent surface and the dye [53].

As ionic strength increases, often due to the addition of salts like NaCl, an inspection effect occurs that reduces the electrostatic attraction between negatively charged dye molecules and positively charged active sites on the surface of the adsorption material. This can often result in a decrease in adsorption capacity. In contrast, in systems where adsorption is predominantly influenced by hydrophobic interactions or π–π stacking, moderate ionic strength may enhance dye removal by reducing repulsion between dye molecules, thereby facilitating a multilayer configuration. Consequently, the influence of ionic strength is complex and tailored upon the characteristics of the adsorbent surface and the prevailing interaction mechanisms [54].

#### 2.2.6. Effect of Temperature and Thermodynamic Functions

Temperature has a significant influence on the adsorption process, affecting adsorption capacity, kinetics, and mechanism. Its effect also varies depending on whether the process is chemical or physical. Figure 7F illustrates the impact of changing temperature on the adsorption of CR dye onto biogenic P/Al_2_O_3_-NPs. Analyzing the results in Figure 7F reveals that temperature negatively affects the removal of CR dye and the adsorption capacity of the biogenic adsorbent. As temperature rises from 293 K to 323 K, both the removal percentage and adsorption capacity decline from 97.3% and 29.3 mg/g to 92.1% and 27.6 mg/g, respectively. This behavior is characterized as physisorption, driven by weak van der Waals forces. This process is typically exothermic due to the increased kinetic energy that promotes desorption. Recent results indicate that the adsorption process between the CR dye molecules and the active sites on the surface of biogenic P/Al_2_O_3_-NPs tends to be exothermic. Chemical adsorption can be described as an endothermic process that involves the formation of strong chemical bonds. In many instances, increasing the temperature has enhanced the adsorption process on active surfaces by supplying the energy required to overcome the energy barrier necessary for bond formation.

To understand the extent of the temperature impact on the adsorption process, it is essential to compute thermodynamic functions for a comprehensive understanding of this temperature-related parameter. Thermodynamic functions such as enthalpy (ΔH°) and entropy (ΔS°) can be calculated by applying the van’t Hoff equation [55],

(1) $$\ln\;K_{d}=\frac{∆S{}^{\circ}}{R}-\frac{∆H{}^{\circ}}{RT}$$

while Gibbs free energy (ΔG°) is calculated by the equation [56]

(2) $$∆G{}^{\circ}=-RT\;\ln{\;K}_{d}$$

(3) $$K_{d}=\frac{q_{e}}{C_{e}}$$

**Figure 8:**
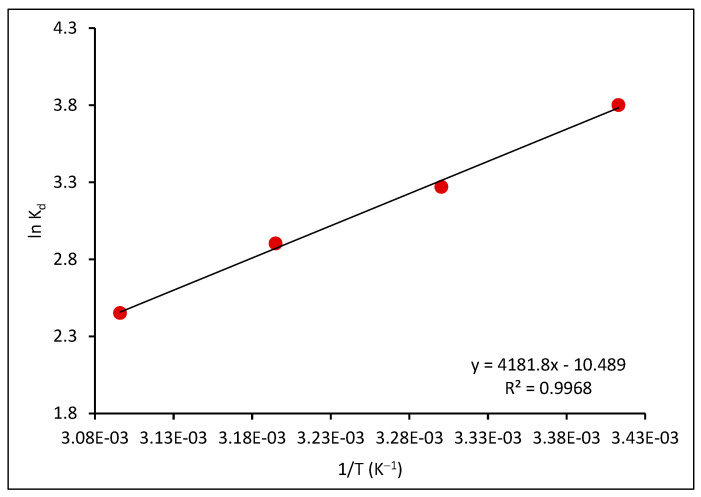
The van’t Hoff plot for adsorption of CR dye onto biogenic P/Al_2_O_3_-NPs derived from *P. pavonica* using waste pharmaceutical packages as precursors.

Here, R stands for the universal gas constant (8.314 J/K mol), and T is the temperature on the Kelvin scale, while K_d_ is the thermodynamic equilibrium constant. Table 3 summarizes the thermodynamic parameters computed from Figure 8 for CR dye adsorption onto biogenic P/Al_2_O_3_-NPs. The ΔG° values ranging from −9.258 to −6.581 kJ/mol confirm that the adsorption of CR dye occurred through a physical mechanism, as ΔG° values below 20 kJ/mol are characteristic of physisorption [57]. The negative values of the Gibbs function indicate that the adsorption process of dye molecules on the surface of biogenic P/Al_2_O_3_-NPs is both spontaneous and favorable [58]. The observed negative values of both ΔG° and ΔH° confirm the exothermic nature of CR dye adsorption onto biogenic P/Al_2_O_3_-NPs, while the concomitant negative ΔS° reflects a decrease in system randomness. Comparable thermodynamic behavior has been reported in several studies using natural waste-based adsorbents or nanocomposites for Congo red removal from aqueous solutions [15,59,60,61].

#### 2.2.7. Kinetic Studies

The interaction of CR dye molecules as an adsorbate with biogenic P/Al_2_O_3_-NPs derived from *Padina pavonica*, using waste pharmaceutical packages as precursors for the adsorbent, can be articulated through several kinetic models. The objective of the adsorption rate studies was to establish an effective model that clarifies the relationship between dye uptake rate and time, facilitating the estimation of retention time to ascertain the optimal amount of adsorbent. The removal of CR over biogenic P/Al_2_O_3_-NPs was determined by fitting data into the following kinetic models.

(4) $$\mathrm{Pseudo}-\mathrm{first}\;\mathrm{order}(\mathrm{Lagergren}\;\mathrm{model}):\;\;\;\;\ln\left(q_{e}-q_{t}\right)=\ln q_{e}-K_{1}t$$

(5) $$Pseudo-second\;order:\;\;\;\;\;\;\;\;\;\;\frac{t}{q_{t}}=\frac{1}{K_{2}\cdot {q_{e}}^{2}}+\frac{1}{q_{e}}t$$

(6) $${Intraparticle\;diffusion:\;\;\;\;\;\;\;\;\;\;q}_{e}=K_{ip}t^{0.5}+C_{i}$$

where q_t_: the amount of adsorbate adsorbed at time t (mg/g), $q_{e}$: the amount of adsorbate adsorbed at equilibrium time (mg/g), $K_{1}$: the pseudo-first-order rate constant (h^−1^) or (min^−1^), $K_{2}$: the pseudo-second-order rate constant (g/mg h) or (g/mg min). The rate constants $K_{1}$, $K_{2}$, $K_{ip}$ can be obtained by a linear plot of ln ( $q_{e}-q_{t}$), t/ $q_{t}$, $q_{e}$ against time, respectively. Table 4 lists the data obtained by applying these selected models. The results indicate that the adsorption of CR dye by P/Al_2_O_3_-NPs follows pseudo-second-order kinetics, with an R^2^ coefficient of 0.999, exceeding the value for pseudo-first-order kinetics. Furthermore, the value of the q_e_ has varied considerably from the experimental measurement. In contrast, the q_e_ value derived from the pseudo-second-order equation was significantly closest to the experimentally obtained value.

#### 2.2.8. Adsorption Isotherms

**Figure 9:**
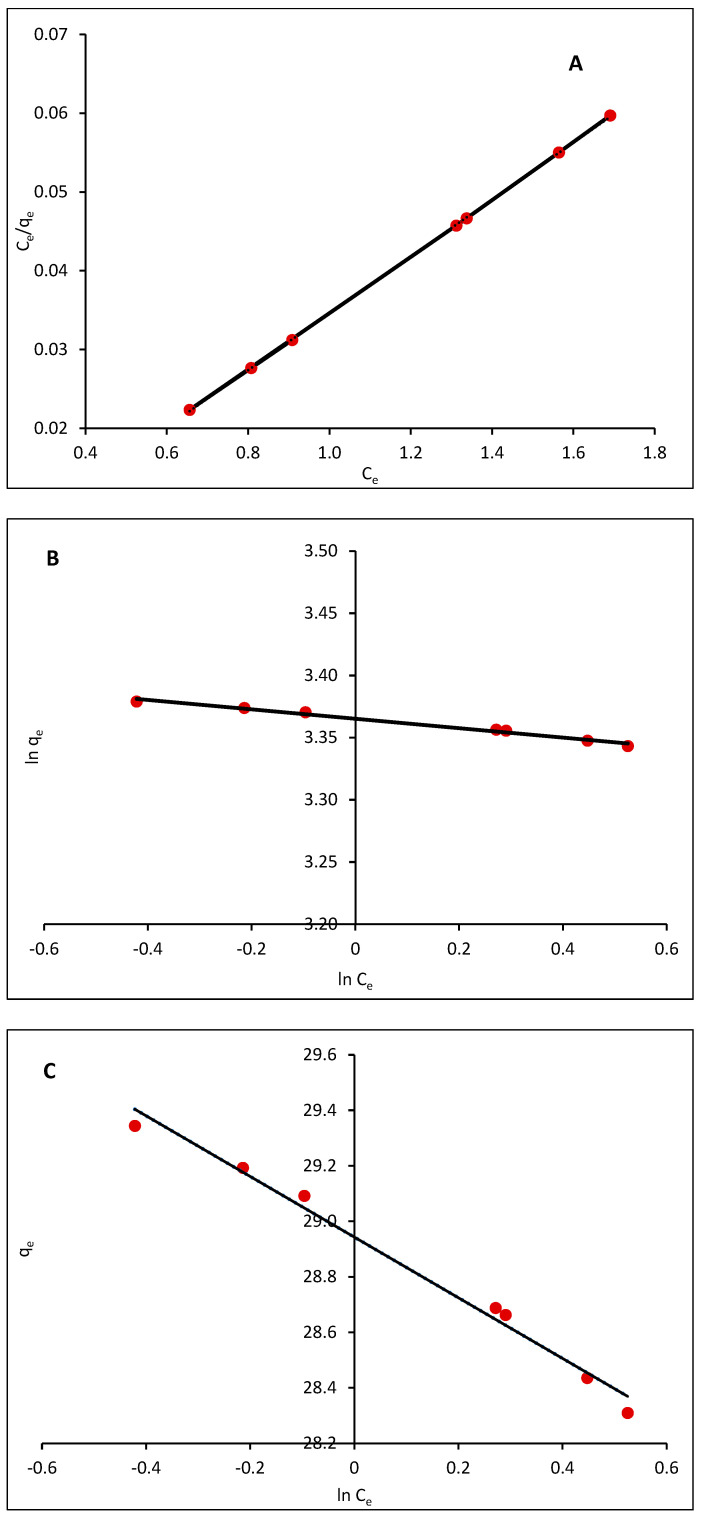
Isotherm models for the adsorption of CR dye onto biogenic P/Al_2_O_3_-NPs derived from *P. pavonica* using waste pharmaceutical packages as precursors: Langmuir model (**A**); Freundlich model (**B**); Temkin model (**C**).

Adsorption isotherm studies are essential because they illustrate the distribution of adsorbed molecules between the adsorbent and liquid solutions at equilibrium. It is fundamental to identify the most appropriate correlation for equilibrium data and empirical equations, as this plays a crucial role in analyzing and designing adsorption systems [62]. The Langmuir, Freundlich, and Temkin models were used to evaluate the data and determine which isothermal model best represents the adsorption of CR over biogenic P/Al_2_O_3_-NPs. The linear forms of the aforementioned isotherm models, along with their corresponding parameter values, as analyzed in Figure 9, are presented in Table 5.

From the results in Table 5, it is clear that the Langmuir model can be successfully applied to visualize the adsorption process of CR dye on the surface of the studied nanocomposite. Preference is given to these models based on the correlation coefficient (R^2^ = 0.999) and maximum adsorption capacity (q_max_ = 27.778 mg/g), which provided compatible results. This isotherm assumes that the adsorption of species occurs on a defined number of identical, equivalent, and homogeneous active sites with uniform surface energy, leading to the formation of a monolayer. The model assumes the absence of subsequent lateral interactions among adjacent adsorbent species [63]. In contrast, the Freundlich model proposes that the adsorption process occurs on a heterogeneous surface with multiple active sites. Consequently, dye molecules are expected to bond to the stronger binding active sites of the biogenic P/Al_2_O_3_-NPs, and the binding strength of these sites will decline as additional molecules attach [64]. However, based on the correlation coefficient (R^2^) from Table 5, it can be suggested that the Langmuir isotherm model better fits the Congo red adsorption using Al_2_O_3_-NPs. This was agreed with many different studies for the removal of anionic dyes using different materials [41,65].

### 2.3. Adsorption Mechanism of CR Dye onto Biogenic P/Al_2_O_3_-NPs

**Scheme 2:**
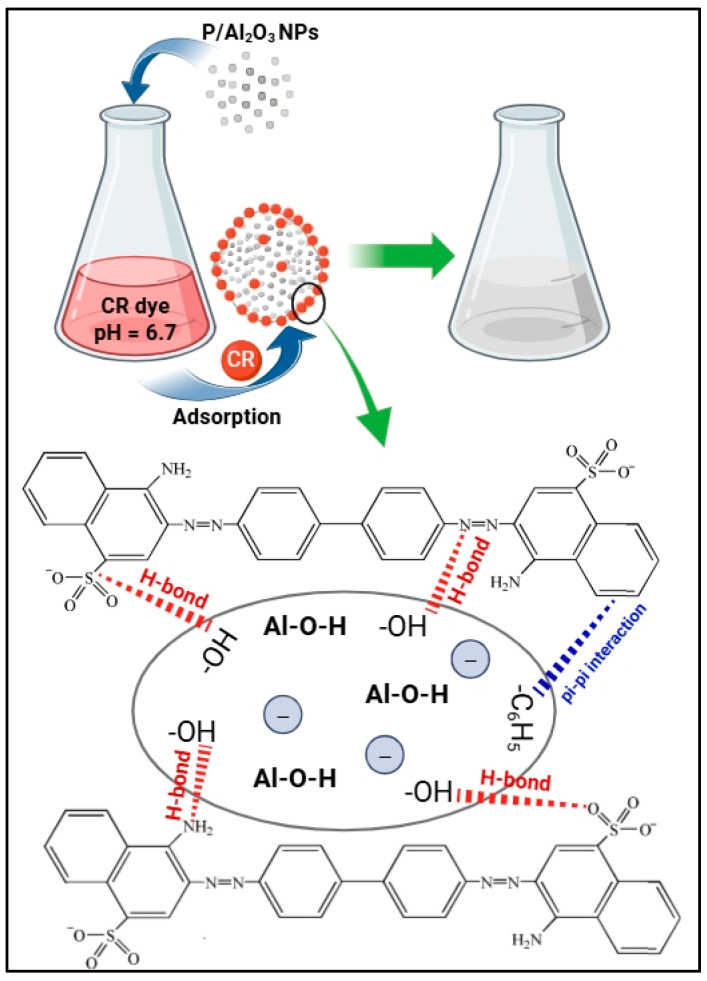
A schematic illustrating the adsorption interaction between CR dye molecules and biogenic P/Al_2_O_3_-NPs.

The results of the current study indicate that biogenic P/Al_2_O_3_-NPs are capable of remediating CR dye molecules via adsorption under ambient conditions. This process is expected to involve numerous and predictable interactions between dye molecules and the nanoparticle surface, including π–π interaction, hydrogen bonding, van der Waals forces, and coordination bonding [42,66]. Considering the variety of these interaction mechanisms, the current adsorption process may proceed through either a single type of interaction or a combination of these phenomena. Among these, π–π interaction may occur between the π-electrons of the biogenic P/Al_2_O_3_-NPs and the aromatic rings of the CR dye molecules. This interaction can further involve charge transfer, polar electrostatic effects, and van der Waals forces [67]. According to zeta potential analysis, the surface of the biogenic P/Al_2_O_3_-NPs carries a negative charge. This negative charge potential is known to enhance nanoparticle stability and significantly influence adsorption behavior. While negatively charged surfaces typically bind efficiently with cationic dyes, the adsorption of anionic dyes such as CR may rely more heavily on non-electrostatic interactions, including hydrogen bonding and other non-electrical interactions. Consequently, hydrogen bond formation is likely between the –NH_2_ groups of the dye and the excess –OH groups on the surface of the biogenic P/Al_2_O_3_-NPs [68,69]. In addition, coordination bonds may form between the azo groups (–N=N–) or amino groups of the dye and the active sites on the P/Al_2_O_3_-NPs [70]. These findings are consistent with previous studies reporting high removal of CR dye using negatively charged nanomaterials, with adsorption capacities exceeding several hundred milligrams per gram of adsorbent [42,71]. Scheme 2 illustrates the proposed adsorption mechanism between CR dye molecules and biogenic P/Al_2_O_3_-NPs.

After CR adsorbs onto biogenic P/Al_2_O_3_-NPs, degradation may occur via surface-driven mechanisms, as shown by earlier research on metal oxide nanoparticles. These materials, especially at the nanoscale, can cause oxidative cleavage of the azo (–N=N–) bond, leading to decolorization and the formation of smaller, less-toxic byproducts [72,73,74,75]. Since Al_2_O_3_ is not highly redox-active, data suggest that surface Al^3+^ centers and oxygen vacancies can promote degradation under UV light, oxidative conditions, or elevated temperatures [76,77]. When CR is adsorbed via hydrogen bonding or π–π stacking, further degradation may occur through radical formation or direct breaking of the azo bond. Consequently, it is probable that the P/Al_2_O_3_-NPs in this study not only effectively adsorb CR dye molecules but may also aid their partial breakdown through surface-catalyzed mechanisms, a concept requiring further investigation.

### 2.4. Comparative Study

The adsorption performance of the biogenic P/Al_2_O_3_-NPs, derived from *Padina pavonica* extract using waste pharmaceutical packages as precursors, was compared with several previously reported adsorbents for CR dye removal (Table 6). The current study found that biogenic P/Al_2_O_3_-NPs possess a maximum adsorption capacity of 27.78 mg/g at an optimal pH of 6.95, with an equilibrium time of 30 min under ambient temperature conditions. This performance aligns with the Langmuir isotherm model, indicating monolayer adsorption behavior. Compared to earlier adsorbents, P/Al_2_O_3_-NPs demonstrate moderate performance, offering significant environmental and operational advantages. These advantages include a short contact time (30 min) for efficient CR removal, biocompatibility, low toxicity in the synthesis process, surface stability, and effective adsorption under near-neutral pH conditions. This positions P/Al_2_O_3_-NPs as a competitive and practical option for CR dye remediation in wastewater treatment applications. However, the reported adsorbents often rely on expensive or toxic precursors and synthetic routes, unlike the green synthesis approach employed in the current study, which utilizes waste materials and marine algae.

## 3. Materials and Methods

### 3.1. Materials

Aluminum pharmaceutical packages were collected as a source of aluminum. The brown marine alga *Padina pavonica* was gathered from the beach in Jeddah, Saudi Arabia, washed, dried at 60 °C, and ground into a fine powder. Congo red (CR), an anionic azo dye (C.I. 22120, MW = 696.68 g/mol), was purchased from Merck (Darmstadt, Germany). The CR dye stock solution (500 mg/L) was prepared in deionized water. Scheme 1 illustrates the chemical structure of this dye. All reagents (HCl and NaOH) were purchased from Merck (Darmstadt, Germany).

### 3.2. Fabrication of P/Al_2_O_3_-NPs

**Scheme 3:**
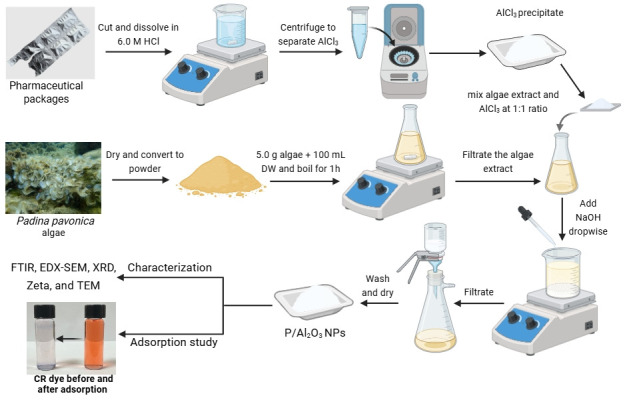
Schematic of the experimental steps for the biogenic fabrication of P/Al_2_O_3_-NPs using waste *Padina pavonica* using waste pharmaceutical packages as precursors.

Approximately 20 g of pharmaceutical packages containing aluminum were cut into small pieces and then dissolved in 200 mL of a 6.0 M HCl solution using magnetic stirring at ambient temperature until the effervescence is removed, forming the aluminum chloride solution. Then, the solution was centrifuged at 10,000 rpm for 30 min, and the supernatant was collected. About 5.0 g of dry marine powder alga *Padina pavonica* was added to 100 mL of distilled water and boiled for 1.0 h. The alga solution was added to the AlCl_3_ solution (1:1), and NaOH was added dropwise until a yellow precipitate forms, which was then filtered, washed, and dried. The functional groups present in biogenic P/Al_2_O_3_-NPs were characterized by Fourier transform infrared spectroscopy (FTIR) using a Frontier spectrophotometer (PerkinElmer, Waltham, MA, USA) with KBr pellet preparation over the 4000–400 cm^−1^ range. Elemental analysis of the biogenic P/Al_2_O_3_-NPs was carried out by field-emission scanning electron microscopy coupled with energy-dispersive X-ray spectroscopy (FE-SEM/EDS; JEOL JSM-6510/V, Tokyo, Japan). Morphological features and particle dimensions of P/Al_2_O_3_-NPs were examined through transmission electron microscopy (TEM; JEOL JSM-6510/V, Tokyo, Japan). Surface charge and colloidal stability were assessed via zeta potential measurements on a Malvern Zetasizer Nano-ZS90 (Malvern, Westborough, PA, USA). Finally, crystalline phases of the P/Al_2_O_3_-NPs were determined by X-ray diffraction (XRD) using a PANalytical X’Pert PRO diffractometer (Spectris plc, Almelo, The Netherlands). Scheme 3 presents a summarized schematic of the experimental steps for the biogenic fabrication of P/Al_2_O_3_-NPs using waste pharmaceutical packages as precursors.

### 3.3. Adsorption Study

Batch biosorption studies were performed to assess the effectiveness of P/Al_2_O_3_-NPs in removing CR dye from aqueous solutions. The adsorption experiments were conducted three times in 50 mL Erlenmeyer flasks, each containing a predetermined dose of P/Al_2_O_3_-NPs and a 10 mL CR dye solution at a specific concentration maintained at 20 °C. The flasks were placed on an orbital shaker set to a shaking speed of 200 rpm. Various conditioning parameters, including pH level, adsorbent dose, contact time, initial concentration, temperature, and ionic strength, were examined. After shaking for the designated time, the sample in the flask was centrifuged for 10 min at 4000 rpm, and the concentration of remaining CR dye in the solution was measured using a Shimadzu UV-1800 spectrophotometer (Shimadzu, Kyoto, Japan) with a 1.0 cm quartz cell in the scan range of 200–800 nm. The adsorption capacity (q_e_) of the biogenic P/Al_2_O_3_ and the removal percentage (RE%) of the CR dye were calculated using the following equations:

(12) $$q_{e}=\left(C_{0}-C_{e}\right)\frac{V}{m}$$

(13) $$RE\%=\frac{C_{0}-C_{e}}{C_{0}}\times 100$$

where V is the volume of the mixture in liters, $C_{0}$ is the initial concentration of the dye, $C_{e}$ is the equilibrium concentration of the dye, and m is the mass of the adsorbent in grams.

#### 3.3.1. Effect of pH

The impact of varying the pH of the solution on the adsorption process was studied by preparing adsorbent–adsorbate systems with different pH levels within the range of 6.18 to 9.98. The systems maintained a fixed initial dye concentration of 30 mg/L and 0.1 g/L of P/Al_2_O_3_ NPs at 20 °C. Adsorption occurred until equilibrium was reached after 30 min with agitation at 200 rpm. It is crucial to ensure that the absorption spectrum of the CR dye remains unaffected by pH variations, thereby allowing for an accurate determination of the removal percentage. In a recent study, at a pH exceeding 10, the dye’s color dissipates, rendering the solution colorless due to changes in the decomposition process. Conversely, at pH values lower than 6, the absorption spectrum of the dye alters due to ionization in acidic media. Several investigations have shown that the CR dye turns dark blue at lower pH [86]. Since the dye was stable within this range, it was chosen as the focus of this study.

#### 3.3.2. Effect of P/Al_2_O_3-_NPs Dosages

To determine the appropriate amount of adsorbent for the subsequent experiments, the effect of varying adsorbent quantities on removal efficiency was examined by preparing adsorbent–adsorbate systems with different amounts of adsorbent (0.25–3.00 g/L) added to a fixed initial dye concentration (30 mg/L). Adsorption was allowed to occur until equilibrium was reached (30 min) at 20 °C with agitation at 200 rpm.

#### 3.3.3. Effect of Contact Time

These experiments involved preparing adsorbent–adsorbate systems using a specific concentration of CR dye (30 mg/L), a dosage of 0.1 g/L of P/Al_2_O_3_-NPs at a specified temperature (20 °C), and applying agitation at 200 rpm over various time intervals (5.0–60 min).

#### 3.3.4. Effect of Initial Concentration of CR Dye

To investigate the effect of changing the concentration of the dye solution on the efficiency of the adsorption process, a set of CR dye concentrations ranging from 5.0 to 50 mg/L was selected for analysis by preparing adsorbent–adsorbate systems with a dosage of 0.1 g/L, at a specific temperature of 20 °C and 200 rpm agitation for an equilibrium time of 30 min.

#### 3.3.5. Effect of Ionic Strength

The effect of ionic strength on the removal of CR dye from aqueous solutions was tested by adding various concentrations of NaCl ranging from 0.4 to 1.0 mol/L to adsorbent–adsorbate systems with a specific dye concentration of 30 mg/L, a dosage of 0.1 g/L, at a temperature of 20 °C, and with agitation at 200 rpm for an equilibrium time of 30 min.

#### 3.3.6. Effect of Temperature

The impact of varying the temperature of adsorbent–adsorbate systems was examined from 20 to 50 °C at a specific concentration of CR dye (30 mg/L), with a dosage of 0.1 g/L and agitation at 200 rpm for a 30 min equilibrium period.

## 4. Conclusions

The environmentally sustainable green method was effectively employed to fabricate biogenic P/Al_2_O_3_-NPs, utilizing pharmaceutical packaging waste as a source of aluminum and marine algae as a reducing and stabilizing agent. The combined results from various characterization techniques (XRD, FTIR, EDX-SEM, TEM, and zeta potential) provided strong evidence for the biogenic fabrication of aluminum oxide nanoparticles. XRD analysis confirmed the high crystallinity of P/Al_2_O_3_-NPs. Meanwhile, SEM and TEM images revealed nanoscale particles ranging in size from 58.63 to 86.70 nm, with morphologies varying from spherical to elliptical. FTIR spectra exhibited characteristic Al–O lattice vibrations at 988 and 570 cm^−1^, supporting the presence of aluminum oxide bonds. Furthermore, the nanoparticles displayed a negative surface charge (−13 mV) and a point of zero charge (pH_pzc_) of 4.8, indicating their stability and potential for electrostatic interaction in aqueous media. The results demonstrated exceptional removal efficiency for CR dye. The effects of CR concentration and pH were investigated to enhance the removal performance of P/Al_2_O_3_-NPs. Batch experimental studies showed that the removal of CR dye using P/Al_2_O_3_-NPs is significantly influenced by the pH of the dye solutions, with the maximum optimal adsorption capacity (q_e_ = 29.344 mg/g) achieved at the natural pH of the CR dye. The equilibrium time for successful adsorption was found to be 30 min. The removal of CR by P/Al_2_O_3_-NPs was analyzed through various kinetic and adsorption models, with optimal fitting obtained via pseudo-second-order kinetics and the Langmuir adsorption isotherm model. The analysis of adsorption thermodynamics indicated that the process was exothermic, and the uptake of CR molecules onto P/Al_2_O_3_-NPs was both feasible and spontaneous. The mechanism of CR adsorption was linked to physiochemical adsorption, as evidenced by pH, isotherm, and FTIR data analyses. It is concluded that the application of P/Al_2_O_3_-NPs for wastewater treatment is both economically feasible and efficient in removing Congo red dye from aqueous solutions. Besides its high efficiency, the developed material shows strong practical potential. The synthesis uses low-cost, sustainable raw materials and can be scaled up with traditional batch or continuous mixing systems. Additionally, the solid adsorbent can be easily incorporated into flow-through columns or modular units for industrial wastewater treatment. These advantages, along with the simplicity and affordability of adsorption technology, support the feasibility of real-world application and technology transfer of the proposed system.

## Figures and Tables

**Table 1:** The element contents of P/Al_2_O_3_-NPs from EDX analysis.

Element	Weight (%)	Atomic (%)
O	13.83	18.76
Na	72.69	70.92
Al	11.00	9.140
Ca	0.680	0.380
Cu	2.250	0.790
Total	100.0	

**Table 2:** X-ray diffraction patterns of biogenic P/Al_2_O_3_-NPs derived from *Padina pavonica* using waste pharmaceutical packages as precursors.

2 Theta	D Value (A)	Intensity (%)	Size (nm)	hkl
27.453	2.246	4.8	82.06	100
30.433	2.935	0.9	82.61	100
31.787	2.819	100	82.89	100
43.527	2.077	0.8	85.83	110
45.519	1.991	87.4	86.45	111
53.932	1.699	2.2	89.44	200
56.537	1.626	11.8	90.51	200
66.280	1.409	9.4	95.20	210

**Table 3:** Thermodynamic functions for CR dye adsorption onto biogenic P/Al_2_O_3_-NPs.

Temperature (K)	ΔG° (kJ mol^−1^)	ΔH° (kJ mol^−1^)	ΔS° (kJ mol^−1^)
293	−9.258	−34.767	−0.0872
303	−8.236		
313	−7.554		
323	−6.581		

**Table 4:** Kinetic parameters for CR dye adsorption onto biogenic P/Al_2_O_3_-NPs.

Pseudo-First Order		Pseudo-Second Order		Intraparticle Diffusion	
q_e_	36.6972	q_e_	29.0710	C_i_	24.9062
K_1_	−0.3361	K_2_	0.0684	K_ip_	0.6043
R^2^	0.8234	R^2^	0.9994	R^2^	0.5124
q_e_ (exp.)	29.3440 mg/g				

**Table 5:** Isotherm equations and constants for CR dye adsorption onto biogenic P/Al_2_O_3_-NPs.

Isotherm	Equations		Parameter Values
Langmuir	C e q e= C e q m a x+ 1 K L q m a x	(7)	q_max_ (mg/g) = 27.778 K_L_ (L/mg) = 24.000 R_L_ = 0.0014 R^2^ = 0.9999
	R L= 1 1+ K L C 0	(8)	
	where $q_{e}$—equilibrium adsorbent capacity, $q_{max}$—maximum adsorbent capacity, $K_{L}$—equilibrium constant of Langmuir, $R_{L}$—dimensionless separation factor.		
Freundlich	ln q e= l n K F+ 1 n l n C e	(9)	K*_F_* (mg/g) = 28.943 *n* = −26.385 R^2^ = 0.985
	where $K_{F}$—Freundlich adsorption constant, $\frac{1}{n}$—heterogeneity factor.		
Temkin	q e= B T l n C e+ B T l n K T	(10)	K*_T_* (L/g) = 3.099 × 10^−12^ B*_T_* (KJ/mol) = −1.093 b*_T_* (mg/g) = −2.229 R^2^ = 0.9864
	B T= R T b T	(11)	
	where $K_{T}$—Temkin isotherm equilibrium binding constant, $B_{T}$—maximum binding heat of sorption, $b_{T}$—binding energy		

**Table 6:** Comparison of P/Al_2_O_3_-NPs performance with other adsorbents for the removal of CR dye.

Adsorbent	q_max_ (mg/g)	%RE	pH	Equilibrium Time	Isotherm Model	Ref.
Doped ZnO	230.0	~99.0	4	10 min	Langmuir	[41]
NiFe_2_O_4_ nanofibers (Ni500)	18.6	97.0	-	30 min	Langmuir	[50]
Coconut husk–raw clay–Fe	1032.0	-	2	480 min	Langmuir	[78]
Pine bark	0.5	94.5	2	7 days	Freundlich	[79]
Kaolinite-supported CoFe_2_O_4_ NPs	547.0	-	-	12 h	Langmuir	[80]
Sol–gel-Cr-doped ZnO NPs	155.5	-	7	110 min	Langmuir	[81]
Al_2_O_3_-ZrO_2_	57.5	80.0	4	60 min	Langmuir	[82]
Biosynthesized ZnO/CuO (BZC)	90.1	-	5	120 min	Langmuir	[83]
Bio-CuO NPs	0.13	99.5	4	80 min	Langmuir	[84]
g-Al_2_O_3_ nanoshells	370.4		7	30 min	Langmuir	[85]
Biogenic P/Al_2_O_3_-NPs	29.3	97.8	6	30 min	Langmuir	This study

## Data Availability

The research data are available.
